# Inhibiting translesion DNA synthesis as an approach to combat drug resistance to DNA damaging agents

**DOI:** 10.18632/oncotarget.17254

**Published:** 2017-04-19

**Authors:** Jung-Suk Choi, Seol Kim, Edward Motea, Anthony Berdis

**Affiliations:** ^1^ Department of Chemistry, Cleveland State University, Cleveland, OH 44115, USA; ^2^ Department of Biological, Geological, and Environmental Sciences, Cleveland State University, Cleveland, OH 44115, USA; ^3^ Departments of Radiation Oncology and Pharmacology, Harold C. Simmons Comprehensive Cancer Center, UT Southwestern Medical Center, Dallas, TX 75390, USA; ^4^ Center for Gene Regulation in Health and Disease, Cleveland State University, Cleveland, OH 44115, USA; ^5^ Case Comprehensive Cancer Center, Cleveland, OH 44106, USA

**Keywords:** DNA damage, DNA polymerization, chemotherapy, nucleoside analogs, leukemia

## Abstract

Anti-cancer agents exert therapeutic effects by damaging DNA. Unfortunately, DNA polymerases can effectively replicate the formed DNA lesions to cause drug resistance and create more aggressive cancers. To understand this process at the cellular level, we developed an artificial nucleoside that visualizes the replication of damaged DNA to identify cells that acquire drug resistance through this mechanism. Visualization is achieved using "click" chemistry to covalently attach azide-containing fluorophores to the ethynyl group present on the nucleoside analog after its incorporation opposite damaged DNA. Flow cytometry and microscopy techniques demonstrate that the extent of nucleotide incorporation into genomic DNA is enhanced by treatment with DNA damaging agents. In addition, this nucleoside analog inhibits translesion DNA synthesis and synergizes the therapeutic activity of certain anti-cancer agents such as temozolomide. The combined diagnostic and therapeutic activities of this synthetic nucleoside analog represent a new paradigm in personalized medicine.

## INTRODUCTION

Cellular DNA is constantly exposed to a wide variety of internal and external DNA damaging agents. While cells possess several pathways to correct damaged DNA, some lesions unfortunately escape repair and their presence can produce devastating cellular effects ranging from mutagenesis and genomic rearrangements to cell death. One conserved mechanism to tolerate unrepaired DNA lesions involves their efficient by-pass in a process termed translesion DNA synthesis (TLS). Since most high-fidelity DNA polymerases involved in chromosomal replication cannot efficiently replicate and bypass damaged DNA, cells rely heavily on the activity of specialized DNA polymerases to accomplish this task [1]. A complete understanding of how these polymerases function at the cellular level has been hindered by the diversity of DNA lesions that form inside a cell coupled with the large number of DNA polymerases that participate in their replication. In humans, for example, at least seven of the 15 different DNA polymerases can replicate structurally diverse DNA lesions such as thymine dimers, abasic sites, and double strand DNA breaks [2–6].

**Figure 1:**
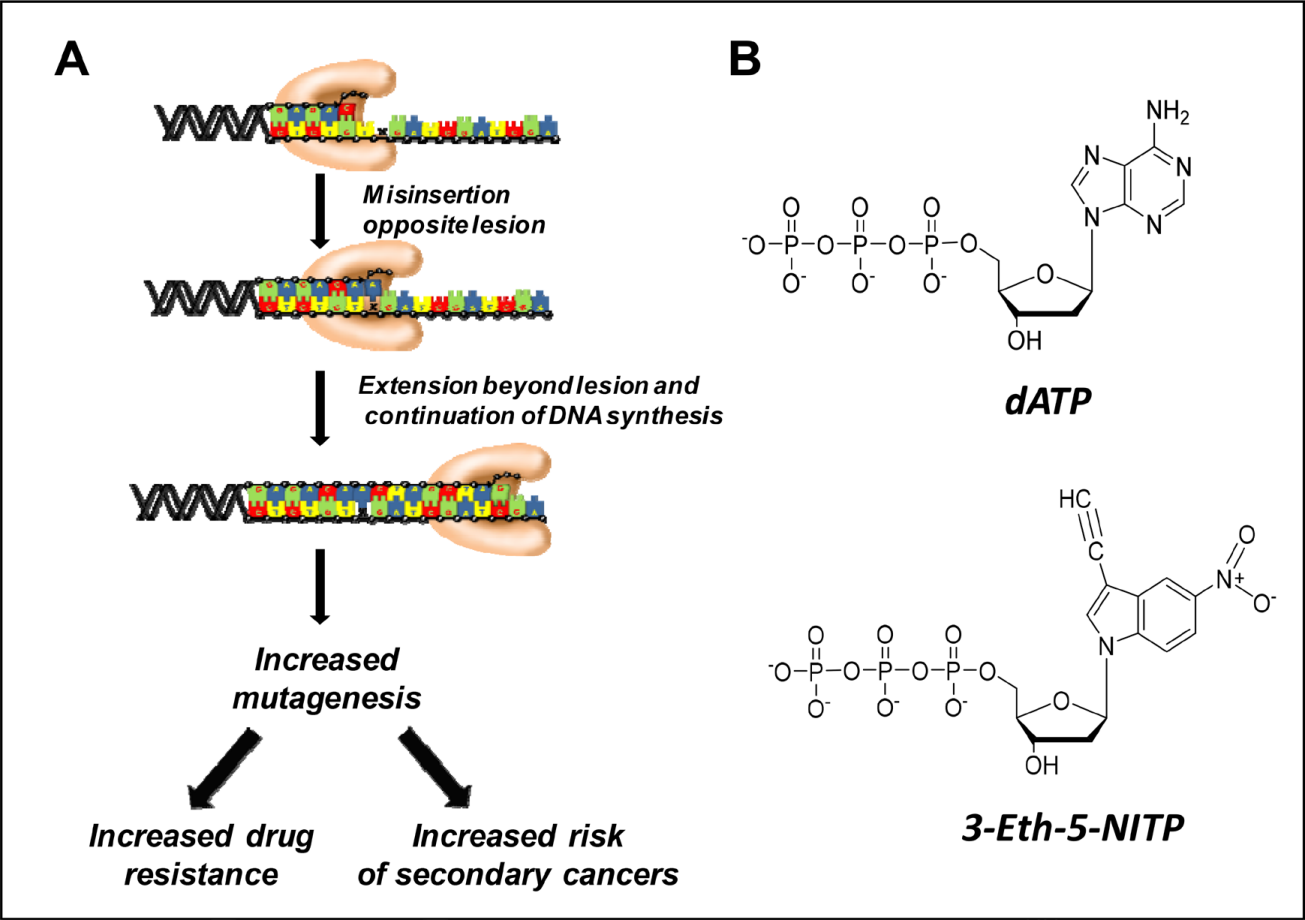
(**A**) Generalized model for translesion DNA synthesis. In this model, a DNA polymerase misinserts a nucleotide opposite a DNA lesion and then extends beyond it. The biological consequences of translesion DNA synthesis include the onset of drug resistance and an increase in mutagenesis. (**B**) Comparison of the chemical structures of dATP and 3-Eth-5-NITP.

Understanding how TLS activity is regulated at the cellular level is especially relevant in cancer patients receiving chemotherapy [7–11]. For example, a significant complication of TLS is the onset of drug resistance caused by misreplicating the DNA lesions produced by agents such as temozolomide (TMZ) and cisplatin [7–9] (Figure 1A). Furthermore, the pro-mutagenic outcomes of TLS can increase the frequency of genetic mutations and create more aggressive cancers. Indeed, Johnson *et al*. recently reported that brain tumors isolated from patients treated with TMZ became drug resistant as a result of acquired somatic mutations in genes associated with DNA mismatch repair [10]. These tumors were hypermutated and contained more than 30 mutations per megabase whereas initial untreated tumors had significantly lower mutation frequencies (0.2 to 4.5 mutations per Mb) [10].

Despite the importance of TLS, there are no chemical compounds that can monitor this process at the cellular level. We have addressed this problem by developing nucleotide analogs that are efficiently and selectively incorporated opposite DNA lesions generated by DNA damaging agents. One therapeutically important DNA lesion is the abasic site which is non-enzymatically formed by DNA alkylating agents such as TMZ and enzymatically by DNA glycosylases [12]. We developed an artificial nucleotide designated 3-ethynyl-5-nitroindolyl-2’-deoxyriboside triphosphate (3-Eth-5-NITP) (Figure 1B) that functions as an efficient surrogate for the natural nucleotide, dATP, that is preferentially utilized during TLS [13]. In this report, we use the corresponding nucleoside, 3-Eth-5-NIdR, to track TLS activity in cancer cells treated with compounds that generate abasic sites. Visualizing the replication of these lesions was achieved using copper-catalyzed “click” chemistry to tag the ethynyl moiety present on the nucleotide with fluorogenic probes. This represents a new diagnostic technique to quantify drug resistance caused by TLS activity. In addition, co-treating leukemia cells with 3-Eth-5-NIdR and anti-cancer agents that generate abasic sites causes a synergistic increase in cell death and correlates with the amount of 3-Eth-5-NITP incorporated into genomic DNA. Collectively, the diagnostic and therapeutic activities of this novel artificial nucleoside provide a new paradigm in personalized medicine for cancer treatment.

## RESULTS

### Measuring the cellular replication of abasic sites

**Figure 2:**
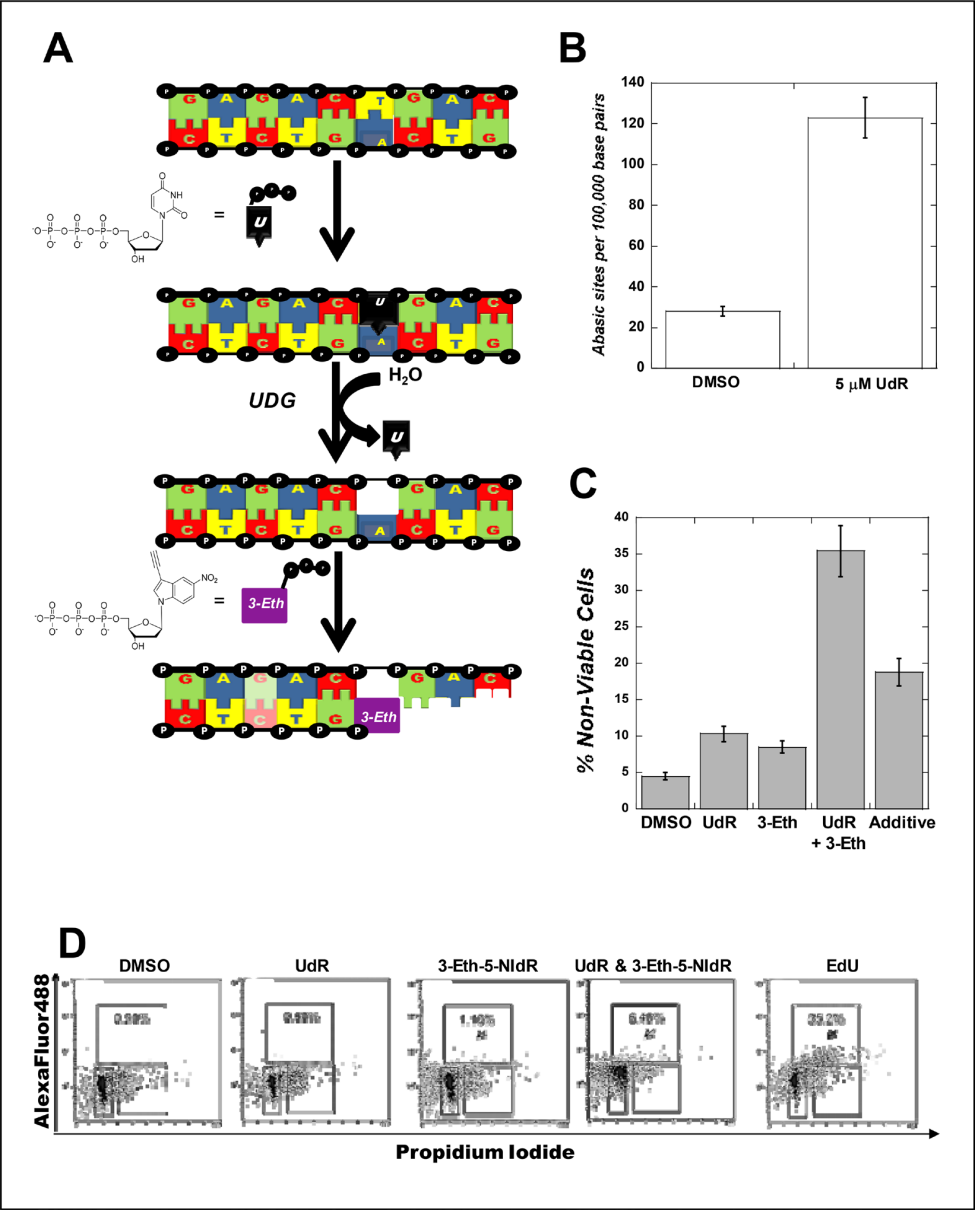
(**A**) Strategy for generating abasic sites under cellular conditions by using uracil DNA glycosylase to excise uracil from DNA in cells treated with UdR. (**B**) Exposure to uracil 2-deoxyribose increases the number of abasic sites in MOLT4 cells. MOLT4 cells were used at an initial density of 200,000 cells/mL and treated with 5 μM uracil 2-deoxyribose (UdR) or DMSO (vehicle control). After 72 hours post-treatment, genomic DNA was isolated, quantified, and diluted in TE buffer to a final concentration of 100 μg/μl. The number of abasic sites in DNA was quantified using the aldehyde reactive probe (ARP) assay. All assays were performed in triplicate, and the means were calculated. Data were calculated on the basis of a linear calibration curve with ARP-DNA standard solution and expressed as number of apurinic sites per 100,000 nucleotides. (**C**) Combining 3-Eth-5-NIdR with UdR generates a synergistic cytotoxic effect compared to treatment with either UdR or 3-Eth-5-NIdR alone. In all cases, the initial density of MOLT4 leukemia cells was maintained at 200,000 cells/mL prior to treatment. Cells were treated with 0.1% DMSO (vehicle), 5 μM UdR, 10 μg/mL 3-Eth-5-NIdR, and 5 μM UdR with 10 μg/mL 3-Eth-5-NIdR for 72 hours. At this time interval, cell viability was assessed using trypan blue staining to count the number of viable versus non-viable cells using a hemocytometer. Cell viability was also assessed with a Muse Cell Count (EMD Millipore). Both assays yield results that are identical within experimental error to each other. (**D**) MOLT4 cells treated with DMSO or 5 μM UdR have low levels (< 0.5%) of AlexaFluor488 labeled DNA while cells treated with 10 μg/mL 3-Eth-5-NIdR have slightly higher levels of AlexaFluor488 labeled DNA (1.1%). Co-treatment with 10 μg/mL Eth-5-NIdR and 5 μM UdR results in a 6-fold increase in AlexaFluor488 labeled DNA (6.4%). Treatment with 10 μM EdU generates considerably higher levels “clicked” genomic DNA (35.2%).

In order to study the cellular replication of damaged DNA, it was necessary to generate abasic sites inside cells. This was achieved by using the enzymatic activity of uracil DNA glycosylase to produce abasic sites by excising uracil from DNA (Figure 2A). In these experiments, MOLT4 leukemia cells were treated with 5 μM uracil 2-deoxyribose (UdR) or DMSO (vehicle control). After 72 hours post-treatment, genomic DNA was isolated and the number of abasic sites in DNA was quantified using the aldehyde reactive probe (ARP) assay [14]. Figure 2B shows that cells treated with 5 μM UdR have a ~4-fold higher number of abasic sites compared to cells treated with DMSO.

The viability of MOLT4 cells treated with 5 μM UdR was also measured using two independent biochemical assays (visualization by microscopy and flow cytometry). Figure 2C shows that cells treated with 5 μM UdR have a ~3-fold higher level of cell death compared to DMSO treatment. Cells treated with 10 μg/mL 3-Eth-5-NIdR (which corresponds to a molar concentration of 33 μM) show a 2-fold increase in cell death. More importantly, cells treated with a low concentration of 3-Eth-5-NIdR (10 μg/mL) and 5 μM UdR show a synergistic increase in cell death that is 2.5-fold higher than the additive effects of UdR and 3-Eth-5-NIdR treatment (Figure 2C). The ability of 3-Eth-5-NIdR to increase the cell killing effects of UdR likely reflects the ability of the corresponding nucleoside triphosphate, 3-Eth-5-NITP, to effectively block the replication of unrepaired abasic sites.

We verified that this synergistic effect is caused by inhibiting TLS activity using dual parameter flow cytometry (propidium iodide (PI) and fluorescence detection of the artificial nucleotide) to measure the amount of AlexaFluor488 covalently attached to 3-Eth-5-NIdR incorporated into genomic DNA. Figure 2D shows that MOLT4 cells treated with DMSO or 5 μM UdR have low levels (< 0.5%) of AlexaFluor488 labeled DNA. Cells treated with 10 μg/mL 3-Eth-5-NIdR for two days display low but appreciable levels of AlexaFluor488 labeled DNA (1.1%). This low level is consistent with previous results demonstrating that 3-Eth-5-NIdR can detect the cellular activity of terminal deoxynucleotidyl transferase (TdT) which is overexpressed in MOLT4 cells [15]. However, cells co-treated with 10 μg/mL Eth-5-NIdR and 5 μM UdR show more robust effects as there is a ~6-fold increase in AlexaFluor488 labeled DNA (6.4%). The increase in “clicked” DNA likely occurs by the incorporation of 3-Eth-5-NITP opposite abasic sites formed after uracil excision. The amount of 3-Eth-5-NIdR utilized during TLS was compared to the amount of 5-ethynyl-2’deoxyribose uracil (EdU) incorporated during normal DNA synthesis (Figure 2D). As expected, cells treated with 10 μM EdU have considerably higher levels “clicked” genomic DNA (35.2%) compared to cells treated with UdR and 3-Eth-5-NIdR (6.4%). In general, the higher amount of "clicked" DNA with EdU treatment results from the efficient insertion of the thymine analog opposite adenine which occurs with a significantly higher frequency in genomic DNA compared to unrepaired abasic sites.

### Specialized DNA polymerases preferentially insert 3-Eth-5-NITP opposite abasic sites

We next investigated which cellular DNA polymerases may be responsible for incorporating 3-Eth-5-NITP opposite abasic sites formed after UdR treatment. *In vitro* approaches measured the kinetic parameters, k_cat_, K_m_, and k_cat_/K_m_, for the utilization of dATP and 3-Eth-5-NITP by pol δ, the high-fidelity polymerase involved in chromosomal replication and pol η, a specialized DNA polymerase that produces drug resistance by replicating damaged DNA [16, 17]. Michaelis-Menten plots for the utilization of dATP by each polymerase are provided as Supplementary Figure 1, and the kinetic parameters derived from these plots are summarized in Table 1. In this analysis, the most important parameter is the k_cat_/K_m_ value as this reflects the overall catalytic efficiency of the polymerase to utilize a nucleotide substrate under physiological conditions. These data indicate that pol δ inserts dATP opposite an abasic site very poorly as the low k_cat_/K_m_ value of 5.5 M^-1^sec^-1^ is caused by a high Km value for dATP (560 ± 180 μM) coupled with a low k_cat_ value (0.0031 ± 0.0004 sec-1). In contrast, pol η is 500-fold more efficient at incorporating dATP opposite the lesion. The high k_cat_/K_m_ value of 2,600 M^-1^sec^-1^ is caused by a 12-fold lower K_m_ value for dATP coupled with a ~40-fold faster k_cat_ value. The observed differences in catalytic efficiencies suggest that pol η is more efficient than pol δ at incorporating dATP opposite abasic sites and thus likely contributes more to the error-prone replication of this lesion under cellular conditions.

**Table 1:** Kinetic parameters for the incorporation of dATP and 3-Eth-5-NITP opposite an abasic site catalyzed by human pol δ and pol η

Nucleotide	Polymerase	K_m_(μM)	k_cat_(sec^-1^)	k_cat_/K_m_(M^-1^sec^-1^)
dATP	pol δ	560 ± 180	0.0031 ± 0.0004	5.5 ± 1.2
dATP	pol η	46 ± 11	0.12 ± 0.01	2,610 ± 550
3-Eth-5-NITP	pol δ	2.0 ± 0.4	0.013 ± 0.001	6,400 ± 900
3-Eth-5-NITP	pol η	3.8 ± 1.2	0.26 ± 0.06	68,420 ± 2,500

Similar experiments were performed using 3-Eth-5-NITP as the substrate (Supplementary Figure 2) and the resulting kinetic parameters are provided in Table 1. In the case of pol δ, the k_cat_/K_m_ value of 6,400 M-1sec-1 for 3-Eth-5-NITP is ~1,200-fold higher than dATP while the catalytic efficiency of ~68,000 M-1sec-1 measured with pol η is ~30-fold higher than dATP. Thus, both high- and low-fidelity DNA polymerases utilize 3-Eth-5-NITP more efficiently than dATP. However, the higher efficiency observed with pol η suggests that specialized polymerases are primarily responsible for utilizing 3-Eth-5-NITP during TLS. Note that exonuclease proofreading activity with this particular nucleotide is extraordinarily low. Thus, the kinetic parameters measured here are not complicated by idle turnover activity and represent an accurate measurement of nucleotide incorporation.

We next examined the ability of both high-fidelity and specialized DNA polymerases to extend beyond dAMP or 3-Eth-5-NIMP paired opposite an abasic site. Both mispairs were formed *in situ* by adding a fixed concentration of nucleotide substrate was added to a solution containing DNA substrate pre-incubated with DNA polymerase. After 4 half-lives, an aliquot of dTTP and dGTP (500 μM final concentration) was added to initiate the elongation reaction. Supplementary Figure 3 provides gel electrophoresis data demonstrating that high-fidelity DNA polymerases such as pol δ and the bacteriophage T4 DNA polymerase efficiently insert 3-Eth-5-NITP opposite an abasic site but are unable to elongate beyond the artificial nucleotide when supplied with natural dNTPs. These results validate the chain termination capabilities of this artificial nucleotide.

Results obtained using pol η (provided as Supplementary Figure 4) are more complicated as the specialized DNA polymerase shows a unique ability to elongate one nucleotide beyond 3-Eth-5-NIMP when paired opposite an abasic site. Although pol η can elongate one base beyond the lesion, it is unable to continue primer elongation when supplied with high concentrations (> 500 μM) of natural dNTPs. Similar behavior is observed when pol η is supplied with dATP. In this case, the specialized DNA polymerase incorporates the artificial nucleotide opposite the lesion and also extends one nucleotide beyond the abasic site. However, pol η possesses significantly higher activity toward elongating beyond dAMP when supplied with natural dNTPs. This activity contrast data obtained with 3-Eth-NIMP which hinders extension beyond the DNA lesion. These results validate that the synthetic analog is a chain terminator of TLS whereas lesion by-pass can more easily occur with natural nucleotides. Collectively, these data validate that the artificial nucleotide analog likely induces cell death by inhibiting the by-pass of abasic sites catalyzed by either high-fidelity or specialized DNA polymerases.

### Measuring translesion DNA synthesis activity in response to chemotherapeutic agents

**Figure 3:**
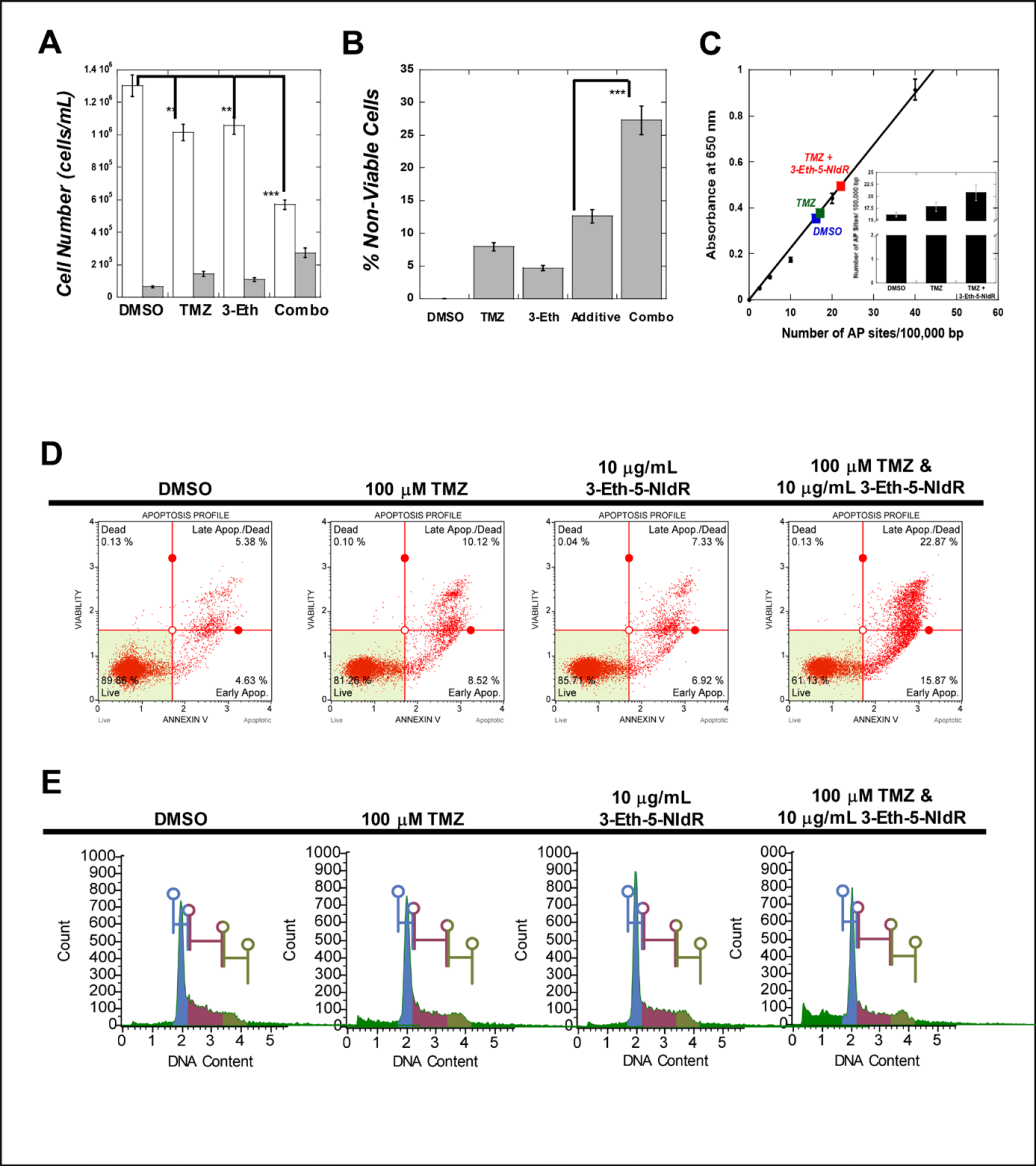
(**A**) Combining sub-lethal doses of 3-Eth-5-NIdR with TMZ produces greater cytostatic and cytotoxic effects compared to treatment with 100 μM TMZ or 10 μg/mL 3-Eth-5-NIdR alone. In all cases, the initial density of MOLT4 leukemia cells was maintained at 200,000 cells/mL prior to treatment. Cells were treated with 0.1% DMSO (vehicle), 100 μM TMZ, 10 μg/mL 3-Eth-5-NIdR, and 100 μM TMZ with 10 μg/mL 3-Eth-5-NIdR for 72 hours. At this time interval, cell viability was assessed using trypan blue staining and flow cytometry as described in the text. Open (white) bars represent viable cells while gray bars represent non-viable cells. ** represents a *p* value of > 0.01 while *** represents a *p* value of > 0.001. (**B**) Secondary plot based on primary data provided in Figure 2A comparing the % non-viable cells as a function of various drug treatments. In all cases, the values representing percent apoptosis caused by various treatment are normalized for cell death measured in the presence of DMSO (vehicle control). This analysis demonstrates that combining 3-Eth-5-NIdR with TMZ produces a 27.5% increase in the percentage of non-viable cells compared to increases of 7.5% and 4.4% with 100 μM TMZ or 10 μg/mL 3-Eth-5-NIdR, respectively. Thus, the combination of 3-Eth-5-NIdR with TMZ generates a synergistic increase in apoptosis as it is greater than the predicted additive effects. *** represents a *p* value of > 0.001 (**C**) Combining 3-Eth-5-NIdR with TMZ increases the number of abasic sites. MOLT4 cells were used at an initial density of 200,000 cells/mL and treated with DMSO (vehicle control), 100 μM TMZ, or 100 μM TMZ with 10 μg/mL 3-Eth-5-NIdR. After 72 hours post-treatment, genomic DNA was isolated, quantified, and diluted in TE buffer to a final concentration of 100 μg/μl. The number of abasic sites in DNA was quantified using the aldehyde reactive probe (ARP) assay. All assays were performed in triplicate, and the means were calculated. Data were calculated on the basis of a linear calibration curve with ARP-DNA standard solution and expressed as number of apurinic sites per 100,000 nucleotides. (**D**) MOLT4 cells treated with 3-Eth-5-NIdR and TMZ have significantly higher levels of early and late stage apoptosis compared to cells treated with TMZ or 3-Eth-5-NIdR alone. Cells (100,000 – 200,000 cells/mL) were treated with 0.1% DMSO (vehicle), 100 μM TMZ, 10 μg/mL 3-Eth-5-NIdR, and 100 μM TMZ with 10 μg/mL 3-Eth-5-NIdR for 48 hours. Cells were harvested by centrifugation, washed in PBS, and re-suspended in 100 μL of binding buffer containing 5 μM of Annexin V-Alexa Fluor 488 conjugate. Cells were treated with 1 μg/ mL PI and incubated at room temperature for 15 min followed by flow cytometry analysis. Cells were analyzed using either Muse Cell analyzer or Beckman Coulter EPICS-XL with EXPO 32 Data Acquisition software. 15,000-gated events were observed for each sample. (**E**) Analysis of cell-cycle progression in MOLT4 treated with TMZ in the absence and presence of 3-Eth-5-NIdR. Cells (100,000–200,000 cells/mL) were treated with 0.1% DMSO (vehicle), 100 μM TMZ, 10 μg/mL 3-Eth-5-NIdR, and 100 μM TMZ with 10 μg/mL 3-Eth-5-NIdR for 48 hours. Cells were harvested by centrifugation, washed in PBS, and treated with 1 μg/ mL PI. Cells were incubated at room temperature for 15 min followed by flow cytometry analysis. Cells were analyzed using either Muse Cell analyzer or Beckman Coulter EPICS-XL with EXPO 32 Data Acquisition software. 15,000-gated events were observed for each sample.

We next assessed the ability of 3-Eth-5-NIdR to potentiate the cell killing effects of TMZ, an anti-cancer agent that generates abasic sites via alkylation of the N^7^-position of guanine [18]. Cell viability was directly compared in cells treated with DMSO, 100 μM TMZ, 10 μg/mL 3-Eth-5-NIdR, and 100 μM TMZ combined with 10 μg/mL 3-Eth-5-NIdR. Figure 3A shows that treatment with either TMZ or 3-Eth-5-NIdR alone for three days produces weak cytostatic and cytotoxic effects. However, more significant effects are observed when TMZ is combined with a sub-lethal dose of 3-Eth-5-NIdR as the number of viable cells decreases with a concomitant increase in the number of non-viable cells. The data provided in Figure 3B normalizes the percentage of non-viable cells as a function of drug exposure against treatment with DMSO to more easily visualize the synergistic effects caused by combining 3-Eth-5-NIdR with TMZ. As illustrated, combining 3-Eth-5-NIdR with TMZ produces a synergistic increase in cell death (27.5%) compared to the additive effects of 11.9% from cells treated individually with 100 μM TMZ (7.5%) or 10 μg/mL 3-Eth-5-NIdR (4.4%).

To verify that this synergistic effect reflects the inhibition of TLS activity, we quantified the number of abasic sites produced by TMZ treatment using the aldehyde reactive probe (ARP) assay [14]. As illustrated in Figure 3C, MOLT4 cells treated with 100 μM TMZ have a 15% increase in the number of abasic sites compared to DMSO treatment. The number of abasic sites formed with 3-Eth-5-NIdR with treatment is identical, within experimental error, to the number of abasic sites produced using DMSO. For convenience, this data point has been omitted from Figure 3C. More importantly, however, we observe that combining 3-Eth-5-NIdR with TMZ produces a 35% increase in the number of abasic sites. This increase could result from two interrelated effects. First, incorporation of 3-Eth-5-NITP opposite an abasic site inhibits the ability of a DNA polymerase to extend beyond the lesion. Secondly, the incorporation of 3-Eth-5-NITP may hinder the efficient repair of abasic sites formed by TMZ treatment. Note that this inhibition does not immediately cause cell death as cells likely attempt to repair stalled replication forks before undergoing apoptosis. Regardless, the combined effects of the inhibition of DNA repair and TLS activity is consistent with the synergistic cell-killing effects caused by combining 3-Eth-5-NIdR with TMZ.

We also tested the ability of 3-Eth-5-NIdR to potentiate the cell killing effects of other anti-cancer drugs such as cisplatin, chlorambucil, carmustine, doxorubicin, and hydroxyurea. These agents were chosen as they produce DNA lesions that are structurally distinct from abasic sites [19–23]. Data provided in Supplementary Table 1 shows that co-treatment with 10 μg/mL 3-Eth-5-NIdR does not increase the cell killing effects of anti-cancer agents that form DNA crosslinks (cisplatin, chlorambucil, and carmustine) or single-strand DNA (hydroxyurea). However, treatment with a sub-lethal concentration of 3-Eth-5-NIdR increases the cytotoxic effects of doxorubicin by 2-fold, and this could reflect the ability of 3-Eth-5-NITP to inhibit the ability of TdT to replicate DSBs formed by doxorubicin [15]. Collectively, these data coupled with the inability of 3-Eth-5-NIdR to potentiate the cytotoxic effects of crosslinked or alkylated DNA lesions indicate that the artificial nucleoside selectively inhibits TLS activity against non-instructional DNA lesions. i.e., abasic sites and DSBs.

### Inhibiting TLS activity increases apoptosis

The cellular mechanisms accounting for the synergistic cell killing effects caused by combining 3-Eth-5-NIdR with TMZ were interrogated using PI uptake and annexin V staining to distinguish live cells from those undergoing early and late stage apoptosis or necrosis. Representative data provided in Figure 3D show that MOLT4 cells co-treated with 3-Eth-5-NIdR and TMZ have significantly higher levels of early and late stage apoptosis (15.9% and 22.9%, respectively) compared to cells treated with TMZ (8.5% and 10.1%, respectively) or 3-Eth-5-NIdR (6.9% and 7.3%, respectively). Values provided in Table 2 represent an average of three independent determinations and are normalized for the effects of DMSO used as the co-solvent. As indicated, the net apoptotic effect (Δ = 28.7%) for combining 3-Eth-5-NIdR with TMZ is 1.6-fold greater than the additive effects of TMZ or 3-Eth-5-NIdR treatment (Δ = 18.2%).

**Table 2:** Summary of dual parameter flow cytometry measuring apoptosis in MOLT4 cells

Condition	Viable	Early Apoptotic	Late Apoptotic	Necrotic	Total Apoptotic
DMSO	90.0 ± 2.3%	4.4 ± 0.8%	4.8 ± 0.5%	0.8 ± 0.1%	9.2 ± 0.6% (0%)
100 μM TMZ	78.5 ± 1.8%	11.0 ± 1.1%	10.3 ± 0.9%	0.2 ± 0.1%	21.3 ± 1.0% (12.1%)
10 μg/mL 3-Eth-5-NIdR	84.3 ± 2.1%	7.7 ± 1.0%	7.6 ± 0.5%	0.4 ± 0.1%	15.3 ± 0.7% (6.1%)
Combination	61.5 ± 1.5%	15.5 ± 1.1%	22.4 ± 0.9%	0.6 ± 0.1%	37.9 ± 1.0% (28.7%)

Values represent an average of three (3) independent determinations performed on different days. Values in parenthesis represent the difference in percent apoptosis of treatment compared to treatment with DMSO (vehicle control).

The effect of this drug combination on cell cycle progression was next examined using PI staining to measure cellular DNA content. Representative histograms are provided in Figure 3E and a summary of three independent determinations are provided in Table 3. A baseline in cell-cycle progression was established by first examining cells treated with DMSO. The histogram displays a pattern consistent with an asynchronous cell population as the majority of cells exist at G0/G1 (40.0 ± 3.2%) with smaller populations at S-phase (27.1 ± 2.5%), G2/M (25.0 ± 3.1%), and sub-G1 (7.9 ± 1.4%). Treatment with 10 μg/mL 3-Eth-5-NIdR over a three day period produces a similar profile (G0/G1 = 40.8 ± 3.9%, S-phase = 23.1 ± 3.2%, G2/M =21.5 ± 2.1%, and sub-G1= 14.6 ± 1.9%). Treatment with 100 μM TMZ also generates a negligible effect on the population of cells at G0/G1 (41.0 ± 4.1%). However, treatment with the DNA damaging agent produces small reductions in cell populations corresponding to S-phase (19.8 ± 1.5%) and G2/M (21.4 ± 2.4%) that occur concomitant with an increase in sub-G1 cells (17.8 ± 2.5%). This increase is consistent with the induction of apoptosis caused by DNA damage after TMZ treatment. More importantly, combining 3-Eth-5-NIdR with TMZ produces higher levels in sub-G1 DNA (30.9 ± 2.9%). This effect appears cell cycle independent as it occurs concomitant with decreases in cell populations at every stage of the cell cycle (G_0_/G_1_ = 34.8 ± 2.5%, S-phase = 18.9 ± 1.9%, and G2M = 15.4 ± 1.5%). The synergistic increase in sub-G1 DNA again suggests that inhibiting lesion by-pass and/or the timely repair of lesions produced by TMZ increases apoptosis.

**Table 3:** Summary of the effects of drug treatment on cell cycle progression in MOLT4 cells

Condition	G0/G1	S-Phase	G2/M	SubG1
DMSO	40.0 ± 3.2%	27.1 ± 2.5%	25.0 ± 3.1%	7.9 ± 1.4% (0%)
100 μM TMZ	41.0 ± 4.1%	19.8 ± 1.5%	21.4 ± 2.4%	17.8 ± 2.5% (9.9%)
10 μg/mL 3-Eth-5-NIdR	40.8 ± 3.9%	23.1 ± 3.2%	21.5 ± 2.1%	14.6 ± 1.9% (6.7%)
Combination	34.8 ± 2.5%	18.9 ± 1.9%	15.4 ± 1.5%	30.9 ± 2.9% (23.0%)

Values represent an average of three (3) independent determinations performed on different days. Values in parentheses represent the difference in percent sub-G1 DNA measured with various treatments compared to treatment with DMSO (vehicle control).

### Using “Click” chemistry to visualize translesion DNA synthesis

**Figure 4:**
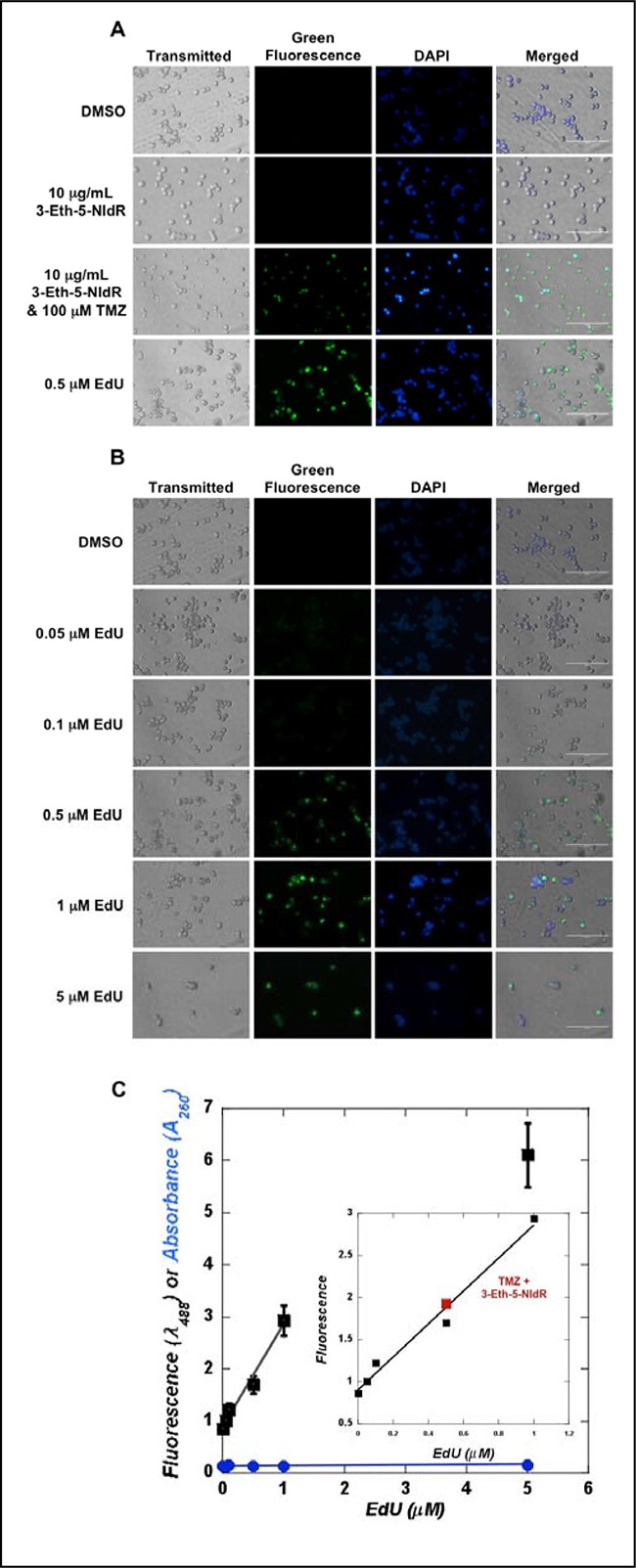
(**A**) Microscopy analyses monitoring the incorporation of 3-Eth-5-NIdR opposite abasic sites generated by TMZ treatment. Cells were co-treated with 100 μM TMZ, 10 μg/mL 3-Eth-5-NIdR, and a combination of 100 μM TMZ and 10 μg/mL 3-Eth-5-NIdR for three days. Cells treated with a combination of 100 μM TMZ and 10 μg/mL 3-Eth-5-NIdR display significantly higher levels of green fluorescence that co-localizes in the nucleus compared to cells treated with 100 μM TMZ or 10 μg/mL 3-Eth-5-NIdR alone. (**B**) Microscopy images of MOLT4 cells treated with increasing concentrations of EdU (0.05 to 5 μM). The fluorescence signal reflecting EdU incorporation increases as the concentration of EdU is raised from 0.05 to 1 μM. See text for experimental details. (**C**) The standard curve for the fluorescence of DNA “clicked” with EdU versus the concentration of EdU is linear (r^2^ = 0.98). DNA isolated from MOLT4 cells treated with 3-Eth-5-NIdR and TMZ (■) shows a level of fluorescence comparable to 0.5 μM EdU. See text for experimental details regarding the generation of the standard curve used to determine the amount of 3-Eth-5-NITP incorporated into genomic DNA.

To verify that 3-Eth-5-NIdR inhibits TLS activity, high-field microscopy techniques were used to visualize the nucleoside in cellular DNA using “click” chemistry to covalently attach fluorogenic probes to the nucleotide incorporated into DNA. Microscopy images provided in Figure 4A show that MOLT4 cells treated with DMSO show insignificant levels of green fluorescence. This negative result is expected since this compound does not contain an alkyne moiety that can react with the azide-containing fluorophore. Cells treated with 10 μg/mL 3-Eth-5-NIdR consistently display slightly elevated levels of green fluorescence. The merged image of green fluorescence with DAPI staining shows nuclear co-localization of the AlexaFluor488 label, indicating that 3-Eth-5-NIdR is incorporated into genomic DNA even in the absence of exogenous DNA damage. However, cells co-treated with 100 μM TMZ and 10 μg/mL 3-Eth-5-NIdR show significantly higher levels of green fluorescence which again co-localizes in the nucleus (panel D). Confocal microscopy images provided as Supplementary Figure 5 validate that the green fluorescence emanating from the “clicked” nucleoside co-localizes within the nucleus. The increased incorporation of 3-Eth-5-NITP into genomic DNA coincides with a higher number of abasic sites produced by TMZ treatment and this provides direct visual evidence for the replication of these DNA lesions inside cells.

We next compared the extent of 3-Eth-5-NITP incorporation during TLS to cells treated with variable concentrations of EdU (0.05 to 5 μM). Images provided in Figure 4B show that the fluorescence signal caused by EdU incorporation increases as the concentration of EdU is raised from 0.05 to 1 μM. Above a concentration of 1 μM, the level of fluorescence appears to plateau, and this may reflect saturation kinetics in the uptake of EdU and/or its metabolism to the corresponding nucleoside triphosphate [24, 25]. Regardless, microscopy images provided in Figure 4A show that cells treated with 3-Eth-5-NIdR and TMZ display fluorescence levels that are similar to cells treated with 0.5 μM EdU. This conclusion was verified by quantifying the amount of fluorescently labeled DNA isolated from cells treated with 3-Eth-5-NIdR and TMZ and comparing it to DNA isolated from cells treated with variable concentrations of EdU (0.05–5 μM). Figure 4C provides a standard curve showing an excellent linear correlation (r^2^ = 0.98) in fluorescence signal as a function of increasing EdU concentrations (from 0.01 to 1 μM). This standard curve was used to determine that DNA isolated from cells treated with TMZ and 3-Eth-5-NIdR displays a level of fluorescence that is comparable to DNA isolated from cells treated with 0.5 μM EdU. Collectively, the results from the microscopy analyses coupled with quantitation of purified DNA from cells verify that 3-Eth-5-NIdR is incorporated opposite DNA lesions generated by TMZ treatment.

## DISCUSSION

TLS is an important biological pathway that provides cells with an effective way to survive genomic stress caused by unrepaired DNA lesions. Unfortunately, this process can also produce detrimental effects at the cellular and organismal levels. Indeed, unregulated TLS activity is involved in the initiation and progression of diseases such as cancer as well as in generating drug resistance to therapeutic agents used to treat this disease. Despite the importance of TLS, a complete understanding of this process has been hindered by the lack of biochemical tools that can directly measure the replication of damaged DNA inside cells. This report addresses this deficiency by using an artificial nucleoside to quantify TLS activity in cells treated with DNA damaging agents. Our results provide key insights into three fundamentally important areas. These include defining the roles of high-fidelity and low fidelity DNA polymerases toward replicating damaged DNA, the development of a diagnostic assay to predict how TLS activity affects therapeutic responses to anti-cancer agents, and a new therapeutic strategy to improve the efficacy of DNA damaging agents used in chemotherapy. Each area is discussed in more detail below.

Currently, there are two accepted models for how DNA polymerase activity is coordinated during TLS [26, 27]. In the first model, a high-fidelity DNA polymerase such as pol δ encounters an unrepaired DNA lesion during chromosomal replication. After incorporating a nucleotide opposite the lesion, the polymerase is unable to extend beyond it. Subsequent stalling of the replication fork the recruits a specialized DNA polymerase such as pol κ or pol ζ to extend beyond the lesion. Once lesion by-pass occurs, pol δ displaces the specialized polymerase and resumes DNA synthesis downstream of the damaged DNA. The second model varies slightly as the intrinsic high-fidelity of pol δ prevents it from incorporating a nucleotide opposite the lesion. Instead, a specialized DNA polymerase such as pol η is recruited to incorporate a dNTP opposite the lesion. After pol η by-passes the lesion, pol δ replaces the specialized polymerase and continues chromosomal replication. In general, the *in vitro* kinetic data provided here are consistent with this second model as pol δ displays low TLS activity as evident in a remarkably low catalytic efficiency for incorporating dATP opposite an abasic site. In contrast, pol η is far more proficient at incorporating dATP and 3-Eth-5-NITP opposite the non-instructional DNA lesion. More importantly, pol η utilizes 3-Eth-5-NITP ~10-fold more efficiently than dATP. This higher efficiency coupled with the chain-termination capabilities of the artificial nucleotide suggests that 3-Eth-5-NITP preferentially inhibits TLS catalyzed by pol η. The inhibitory effects against this specialized DNA polymerase explains how 3-Eth-5-NIdR increases the cytotoxic effects of compounds such as UdR and TMZ that create non-instructional DNA lesions such as abasic sites.

Expanding on these results, we applied “click” chemistry to selectively and covalently attach fluorogenic probes to 3-Eth-5-NIdR to visualize the cellular activity of pol η during TLS. This has important clinical applications, particularly with respect to developing diagnostic tests to determine patient responses to anti-cancer agents that damage DNA. Accurately measuring the effects of chemotherapeutic agents has obvious implications for facilitating successful patient responses to drug treatment. This is especially relevant with therapeutic agents such as cisplatin, doxorubicin, and TMZ which are widely used to treat breast, pancreatic, and brain cancer. To date, most efforts in the area of personalized medicine have focused on using genomic and/or proteomic profiling techniques to identify prognostic biomarkers for therapeutic intervention. Several groups have shown that higher POLH expression correlates with poor patient outcomes, particularly with respect to treatment with DNA damaging agents [28–30]. Unfortunately, similar genetic approaches have failed to produce clear correlations in patient responses to chemotherapy with other specialized DNA polymerases such as pol k and pol z [31–33]. These discrepancies likely reflect the complexities associated with the large number and diversity of human DNA polymerases that can cause drug resistance by replicating damaged DNA. We propose that 3-Eth-5-NITP can overcome these complications since this artificial nucleotide behaves as a universal and highly selective substrate for chromosomal and specialized DNA polymerase that replicate abasic sites. The unique ability of 3-Eth-5-NIdR to directly measure TLS activity against this lesion can be used in activity-based assays to quantify the collective activity of all cellular DNA polymerases that perform TLS. This activity based assay would provide more physiologically relevant data compared to genomic and proteomic techniques which simply infer enzyme activity by measuring mRNA or protein levels.

Finally, the data provided here clearly show the potential therapeutic utility for inhibiting TLS activity as a way to increase the cell killing effects of anti-cancer agents that damage DNA. Our data demonstrate that low concentrations of 3-Eth-5-NIdR significantly increase the cytotoxicity of TMZ by inhibiting TLS activity. This inhibition could produce several beneficial effects in patients receiving chemotherapy. For instance, sensitizing cancer cells to the effects of a DNA damaging agent provides a strategy to administer lower drug doses which would reduce the risk of potential side effects. Again, this is particularly important with drugs such as cisplatin and cyclophosphamide that produce severe and debilitating side effects. Indeed, the ability of the these agents to non-selective kill of healthy yet rapidly proliferating cells such as B- and T-cells accounts for side effects such as leukopenia and thrombocytopenia that can compromise a patient's response to chemotherapy [34]. Finally, targeting TLS activity also provides a rationale way to combat drug resistance caused by the up-regulation of pro-mutagenic DNA synthesis.

## MATERIALS AND METHODS

### Cells and cell culture

MOLT4 cells were cultured in a humidified atmosphere of 5% CO_2_ at 37°C. Cells were maintained in ATCC-formulated RPMI-1640 media supplemented with 10% fetal bovine serum (FBS), 5% L-glutamine, and 0.5% penicillin/streptomycin (Invitrogen, NY).

### Reagents

Phosphate-buffered saline (PBS), antibiotic and antifungal agents, amphotericin, propidium iodide, PrestoBlue, DAPI, Alexa Fluor 488, and apoptosis assay kit containing Alexa Fluor 488-labeled Annexin V were from Invitrogen. 3-Eth-5-NIdR and 3-Eth-5-NITP were synthesized and purified as previously described [13, 15]. DNA including that containing an abasic site were obtained from Operon and purified as described [13, 15]. Recombinant human polymerase delta (pol δ) and human polymerase eta (pol η) were purified as previously described [35, 36]. Each polymerase was judged to be > 97% pure as assessed by sodium dodecylsulfate-polyacrylamide denaturing gel electrophoresis.

### Cell viability assays

3-Eth-5-NIdR was added to wells in a dose-dependent manner (1−100 μg/mL) and treated for variable time periods (24−72 hr). In all cases, the final concentration of the co-solvent, DMSO, was maintained at 0.1%. Cell viability was assessed using trypan blue staining to count the number of viable versus non-viable cells using a hemocytometer. Cell viability was also assessed with a Muse Cell Count (EMD Millipore). IC_50_ values for the artificial nucleoside and anti-cancer agents were obtained using a non-linear regression curve fit of the data to Equation 1.

y = 100% / (1 + (IC_50_/[Compound]))   (1)

LD_50_ values for the artificial nucleoside were calculated using identical approaches.

### Apoptosis measurements

Cells (100,000 – 200,000 cells/mL) were treated with 0.1% DMSO (vehicle), 5 μM UdR, 10 μg/mL 3-Eth-5-NIdR, and 5 μM UdR with 10 μg/mL 3-Eth-5-NIdR for 48 hours. Cells were harvested by centrifugation, washed in PBS, and re-suspended in 100 μL of binding buffer containing 5 μM of Annexin V-Alexa Fluor 488 conjugate. Cells were treated with 1 μg/ mL PI and incubated at room temperature for 15 min followed by flow cytometry analysis. Cells were analyzed using either Muse Cell analyzer or Beckman Coulter EPICS-XL with EXPO 32 Data Acquisition software. 15,000-gated events were observed for each sample.

“Click” reactions were performed using cells harvested after 2 days of treatment with DMSO, EdU (0–5 μM), TMZ (100 μM), 3-Eth-5-NIdR (10 μg/mL), or TMZ (100 μM) with 3-Eth-5-NIdR (10 μg/mL). All cells were fixed with cold methanol overnight. Cells were treated with 0.3 mL of saponin-based permeabilization and wash buffer for 45 min at 37 °C. Click reactions were initiated with click-iT reaction cocktail followed by incubation at 37 °C for 90 min. Cells were washed twice with wash buffer. Cell pellets were dislodged using 0.5 mL solution of 10 μg/mL PI and RNAase A in saponin-based permeabilization buffer. Cells were incubated for 15 min with 1 μg/mL DAPI prior to analysis. Images were obtained using an EVOS_fl_ Advanced microscope (40X magnification).

### Kinetic parameters for nucleotide incorporation

Kinetic studies using polymerase delta and polymerase eta were performed using an assay buffer consisting of 50 mM TrisOAc, 1 mg/mL bovine serum albumin, 10 mM DTT, and 5 mM MgCl_2_ at pH 7.5. All assays were performed at 37°C. *k*_cat_, *K*_m_, and *k*_cat_/*K*_m_ values for nucleotides were measured as described (37). Data for the dependency of rate as a function of nucleotide concentration were fit to the Michaelis–Menten equation (Equation 3):

*ν* = V_max_ * [dXTP] / (K_m_ + [dXTP])   (3)

where *ν* is the rate of product formation analysis, the most important parameter (nM/s), *V*_max_ is the maximal rate of polymerization, *K*_m_ is the Michaelis constant for dXTP, and [dXTP] is the concentration of nucleotide substrate. The turnover number, *k*_cat_, is *V*_max_ divided by the final concentration of polymerase used in each assay.

### Chain-termination experiments

Assays were performed using pseudo-first order reaction conditions in which a limiting concentration of DNA polymerase (25 nM) was pre-incubated with 500 nM DNA containing an abasic site in assay buffer and then mixed with a fixed concentration of dATP (500 μM) or 3-Eth-5-NITP (5 μM) to initiate insertion opposite the lesion. After 4 half-lives, an aliquot of dTTP and dGTP (500 μM final concentration) was added to initiate the elongation reaction. Aliquots of the reactions were quenched with 200 mM EDTA at variable times (0-30 minutes) and analyzed by denaturing gel electrophoresis to assess elongation beyond dATP or 3-Eth5-NITP.

### Quantifying abasic site formation

DNA was isolated using the genomic DNA mini kit as described by the manufacturer (IBI Scientific). The concentration and purity of isolated DNA was measured using agarose gel electrophoresis and spectrophotometric analyses (Spectramax M4, Molecular Devices). Genomic DNA was diluted in TE buffer to a final concentration of 100 μg/μl. Measurements were performed with the use of a commercially available kit for abasic sites site counting (DNA Damage Quantification Kit, Dojindo Molecular Technologies). All assays using the aldehyde reactive probe (ARP) were performed in triplicate, and the means were calculated. Data were calculated on the basis of a linear calibration curve with ARP-DNA standard solution and expressed as number of apurinic sites per 100,000 nucleotides.

### Statistical analyses

All data showing error bars are presented as mean ± s.e.m. The significance of difference in the mean value was determined using a two-tailed Student's *t*-test and normal distribution was assumed in all cases. A one-way ANOVA analysis was used to compare the effects of cells treated with the combination of 3-Eth-5-NIdR and DNA damaging agent versus treatment with DMSO, 3-Eth-5-NIdR, and DNA damaging agent alone to determine *p*-values. *P*-values < 0.05 were considered significant. All calculations were performed using KaleidaGraph software. All cell culture experiments were reproduced at least three times independently. For each experiment, the number of samples and replicates are provided in the text or figure legend.

## SUPPLEMENTARY MATERIALS FIGURES AND TABLES


