# Supplementary material for: Inhibiting translesion DNA synthesis as an approach to combat drug resistance to DNA damaging agents
Source: Oncotarget. 2017 Apr 19;8(25):40804–16. doi: 10.18632/oncotarget.17254 (PMC5522278; doi:10.18632/oncotarget.17254)
Supplement: Supplementary file 1 [file oncotarget-08-40804-s001.pdf]

Inhibiting translesion DNA synthesis as an approach to combat drug resistance to DNA damaging agents

Supplementary Materials

Supplementary Table 1: Summary of LD_{50} values measured for various anti-cancer agents against MOLT4 leukemia cells in the absence or presence of 10 µg/mL 3-Eth-5-NIdR

Compound	LD_{50} value (minus 3-Eth-5-NIdR)	LD_{50} value (plus 3-Eth-5-NIdR)
Cisplatin	21.2 +/− 5.8 µM	20.0 +/− 6.8 µM
Chlorambucil	0.24 +/− 0.03 µM	0.21 +/− 0.05 µM
Carmustine	3.1 +/− 1.0 µM	3.2 +/− 0.9 µM
Doxorubicin	37.5 +/− 12 nM	21 +/− 8 nM
Hydroxyurea	155 +/− 25 µM	150 +/− 30 µM

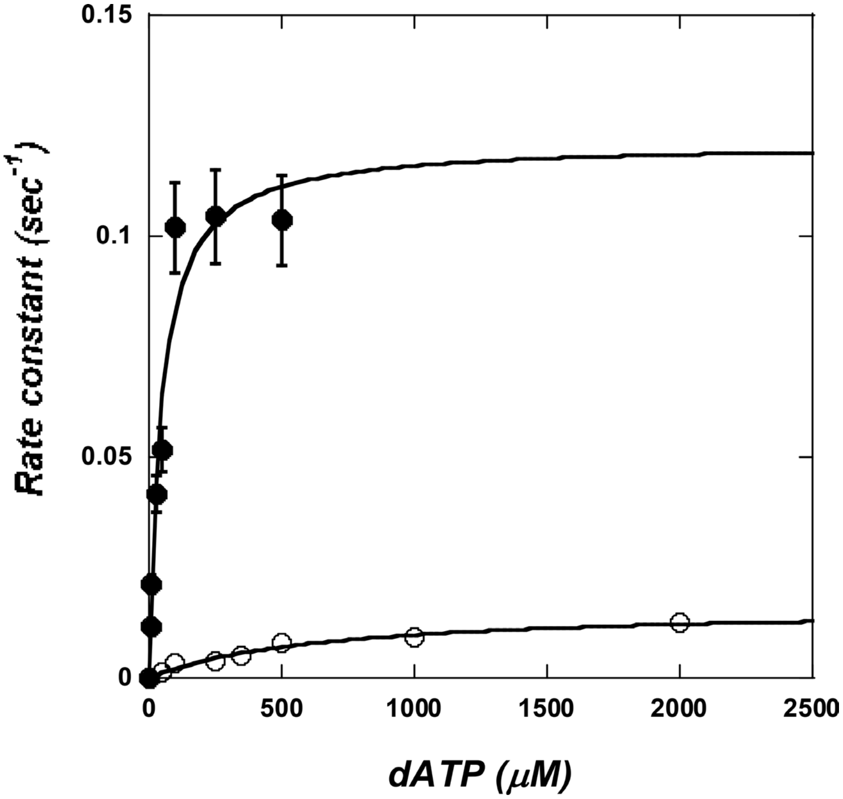

Supplementary Figure 1: Michaelis-Menten plots for the utilization of dATP catalyzed by pol delta (o) and pol eta (●) during the replication of an abasic site. Kinetic studies were performed using an assay buffer consisting of 50 mM TrisOAc, 1 mg/mL bovine serum albumin, 10 mM DTT, and 5 mM $MgCl_2$ at pH 7.5. All assays were performed at 37°C. Data for the dependency of rate as a function of nucleotide concentration were fit to the Michaelis–Menten equation, $v = V_{max} * [dXTP]/(K_m + [dXTP])$, where v is the rate constant of product formation (1/s), V_{max} is the maximal rate constant of polymerization, K_m is the Michaelis constant for dATP, and [dATP] is the concentration of nucleotide substrate.

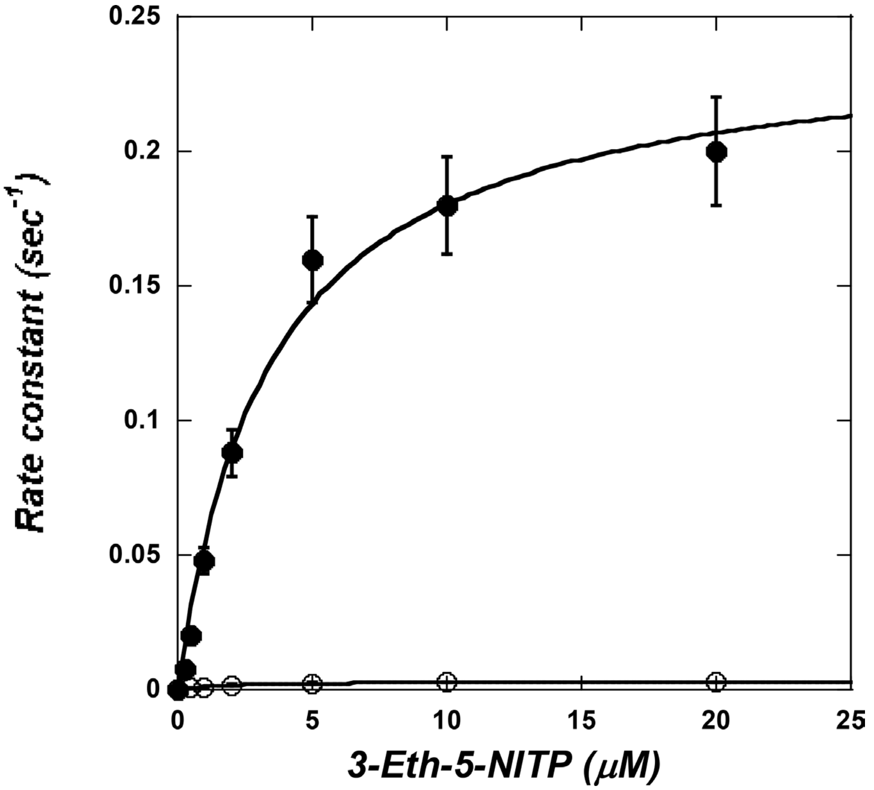

Supplementary Figure 2: Michaelis-Menten plots for the utilization of 3-Eth-5-NITP by pol delta (o) and pol eta (●) during the replication of an abasic site. Kinetic studies were performed using an assay buffer consisting of 50 mM TrisOAc, 1 mg/mL bovine serum albumin, 10 mM DTT, and 5 mM $MgCl_2$ at pH 7.5. All assays were performed at 37°C. Data for the dependency of rate as a function of nucleotide concentration were fit to the Michaelis–Menten equation, $v = V_{max} * [dXTP] / (K_m + [dXTP])$, where v is the rate constant of product formation (1/s), V_{max} is the maximal rate constant of polymerization, K_m is the Michaelis constant for 3-Eth-5-NITP, and [3-Eth-5-NITP] is the concentration of nucleotide substrate.

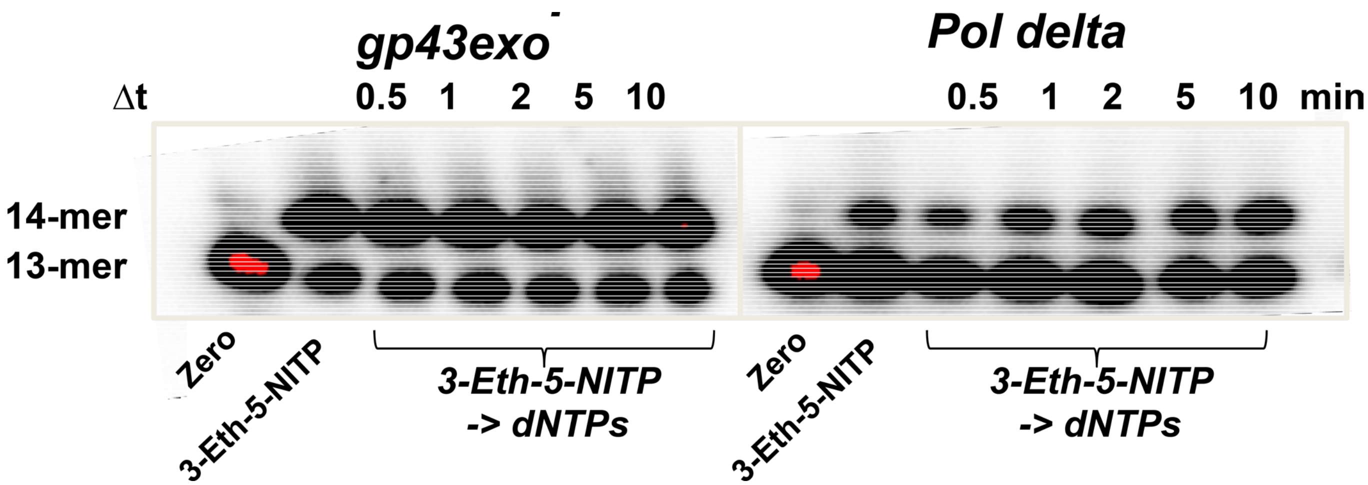

Supplementary Figure 3: Chain-termination capabilities of 3-Eth-NITP against high fidelity DNA polymerases. Assays were performed using pseudo-first order reaction conditions in which a limiting concentration of the bacteriophage T4 DNA polymerase or human pol delta (25 nM) was pre-incubated with 500 nM DNA containing an abasic site in assay buffer and then mixed with a fixed concentration of 3-Eth-5-NITP (5 μM) to initiate insertion opposite the lesion. After 4 half-lives, an aliquot of dTTP and dGTP (500 μM final concentration) was added to initiate the elongation reaction. Aliquots of the reactions were quenched with 200 mM EDTA at variable times (0–30 minutes) and analyzed by denaturing gel electrophoresis to assess elongation beyond 3-Eth-5-NITP.

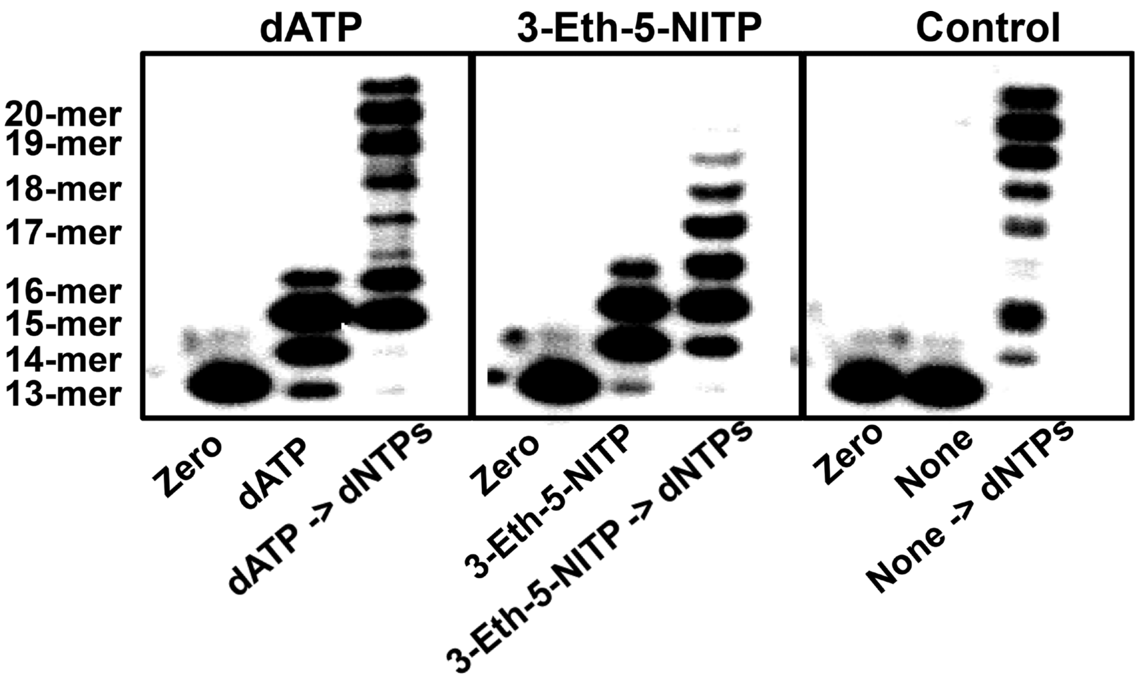

Supplementary Figure 4: Chain-termination capabilities of 3-Eth-NITP against pol eta. Assays were performed using pseudo-first order reaction conditions in which a limiting concentration of human pol eta (25 nM) was pre-incubated with 500 nM DNA containing an abasic site in assay buffer and then mixed with a fixed concentration of dATP (500 µM) or 3-Eth-5-NITP (5 µM) to initiate insertion opposite the lesion. After 4 half-lives, an aliquot of dTTP and dGTP (500 µM final concentration) was added to initiate the elongation reaction. Aliquots of the reactions were quenched with 200 mM EDTA at variable times (0–30 minutes) and analyzed by denaturing gel electrophoresis to assess elongation beyond dATP or 3-Eth-5-NITP.

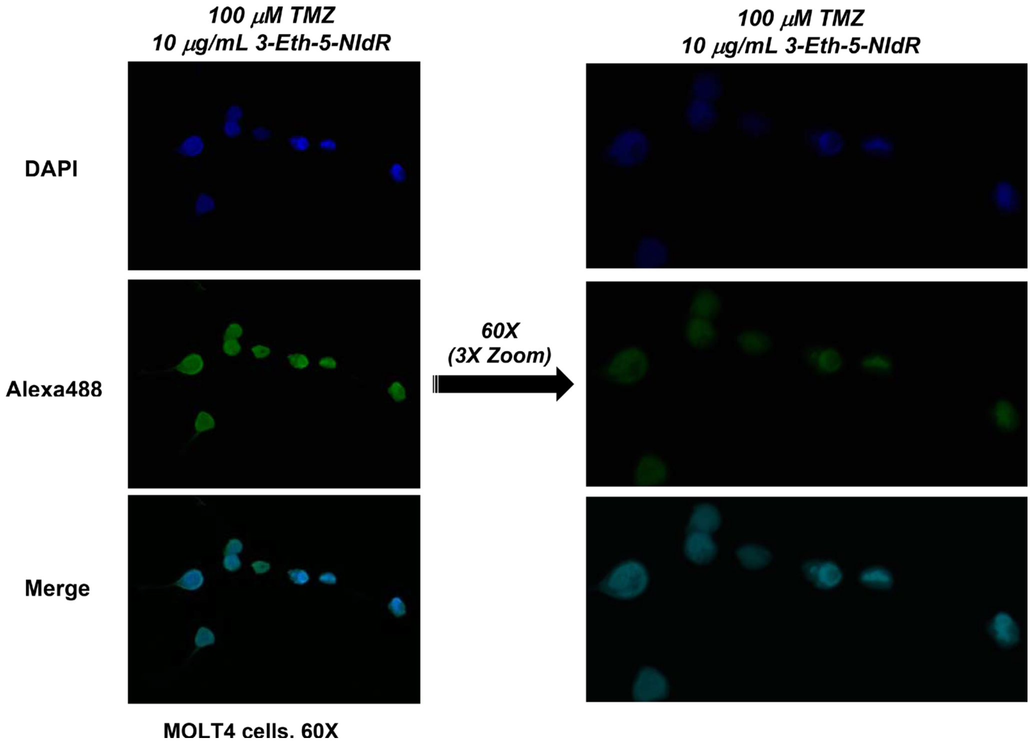

Supplementary Figure 5: Confocal microscopy images of MOLT4 cells treated with 100 µM TMZ and 10 µg/mL 3-Eth-5-NIdR. The merged image of DAPI and Alexa488 shows nuclear co-localization, thus validating the enzymatic incorporation of 3-Eth-5-NITP opposite damaged DNA.
